# When weight-loss drugs reduce drinking: glucagon-like peptide-1 receptor agonists as phenotype-enriched harm-reduction therapy for alcohol use disorder

**DOI:** 10.1097/MS9.0000000000005709

**Published:** 2026-09-21

**Authors:** Sarah Singh, Mehta Das

**Affiliations:** a Department of Internal Medicine, Vita-Salute San Raffaele University, Milan, Italy; b Department of Internal Medicine, Kabul Medical University, Kabul, Afghanistan

**Keywords:** alcohol use disorder, GLP-1 receptor agonist, harm reduction, obesity, semaglutide

## Abstract

Glucagon-like peptide-1 receptor agonists (GLP-1 RAs) have attracted interest as potential treatments for alcohol use disorder (AUD) after patients receiving these agents for obesity or type 2 diabetes reported a reduced desire to drink. This editorial examines whether current evidence supports their classification as broadly applicable addiction treatments or as phenotype-enriched therapies. Randomized trials evaluating exenatide and semaglutide suggest a clinically relevant but endpoint-selective signal. Reductions have been most consistent in heavy-drinking days, drinks per drinking day, naturalistic craving, alcohol-related consequences, and the World Health Organization (WHO) risk drinking level, whereas effects on total alcohol consumption, drinking frequency, and laboratory cue-elicited craving have been less consistent. Durable abstinence, sustained benefit after treatment discontinuation, prevention of alcohol-related hospitalization in randomized trials, and mortality benefit remain unestablished. Obesity may enrich treatment response and provide an independent metabolic indication, but current evidence neither identifies it as a necessary response biomarker nor establishes that alcohol reduction is mediated by weight loss. We therefore propose a three-axis clinical selection framework based on alcohol phenotype, metabolic phenotype, and treatment safety and feasibility. The strongest current rationale may be for patients with heavy episodic or loss-of-control drinking who also have obesity, type 2 diabetes, or substantial cardiometabolic risk. GLP-1 RAs should presently be regarded as candidate harm-reduction therapies that complement established AUD care, not as treatments for acute alcohol withdrawal or universal substitutes for approved pharmacotherapy. Larger comparative trials incorporating mechanistic outcomes and post-discontinuation follow-up are needed.

## Introduction

Glucagon-like peptide-1 receptor agonists (GLP-1 RAs) entered alcohol use disorder (AUD) research after patients treated for diabetes or obesity unexpectedly reported diminished interest in drinking. What began as an anecdote now has convergent human support[1]. An initial randomized trial of exenatide did not improve the primary drinking outcome overall, but reduced alcohol-cue reactivity and yielded an exploratory efficacy signal among participants with obesity[2]. Subsequent semaglutide trials have reported reductions in laboratory alcohol self-administration, craving, heavy-drinking days, drinks per drinking day, alcohol-related consequences, and World Health Organization (WHO) risk-drinking levels, although effects have varied across endpoints^[^3–5^]^. These findings shift the question from whether a biological signal exists to which patients are likely to respond, which outcomes improve, and whether any benefit reflects central reward modulation, weight loss, treatment-related aversion, or their interaction. This editorial synthesizes the endpoint-specific evidence, examines obesity as a mechanism, response marker, or co-indication, and proposes a three-axis selection framework integrating alcohol phenotype, metabolic phenotype, and treatment safety and feasibility. Clarifying these distinctions may prevent premature universal prescribing while identifying patients for whom GLP-1 RA therapy could provide both alcohol-related harm reduction and established metabolic benefit.

A real signal, but an endpoint-selective one.

Across randomized trials, the emerging signal is potentially clinically meaningful but endpoint-selective. The most consistent effects concern drinking intensity and alcohol-related harm rather than complete cessation. Across semaglutide trials, reductions have been most consistent for heavy-drinking days, drinks per drinking day, naturalistic craving, and the likelihood of remaining at a higher WHO risk drinking level^[^3–5^]^. These outcomes matter because movement to a lower WHO risk level is associated with improved health and functioning even when abstinence is not achieved[6]. The 2026 *Lancet* trial provides particularly relevant evidence from a clinically engaged population. Among 108 treatment-seeking adults with AUD and obesity who also received cognitive behavioral therapy, 26 weeks of semaglutide produced a 41.1% reduction in heavy-drinking days, 13.7 percentage points greater than that observed with placebo, alongside improvements in weight and cardiometabolic measures^[^4^]^.

The signal is less consistent for aggregate alcohol consumption and experimentally elicited craving. In the 2025 injectable semaglutide trial, treatment reduced laboratory alcohol self-administration, drinks per drinking day, and weekly craving, but did not reduce drinks per calendar day or the number of drinking days[3]. Similarly, oral semaglutide reduced heavy-drinking days, naturalistic craving, alcohol-related consequences, and WHO risk drinking level, but did not significantly improve the primary laboratory cue-elicited craving endpoint or drinks per day[5]. This pattern suggests that controlled cue paradigms, drinking frequency, consumption intensity, and total volume capture distinct dimensions of treatment response and should not be regarded as interchangeable measures of efficacy.

Important outcomes remain unestablished. No randomized trial has demonstrated durable abstinence, sustained protection after treatment discontinuation, prevention of alcohol-related hospitalizations, or improvements in long-term mortality. Existing trials have limited sample sizes and follow-up durations, and were not designed to determine whether alcohol-related benefits persist after weight regain or treatment withdrawal. GLP-1 RA therapy may therefore function more like a brake on escalation and binge intensity than an abstinence-producing switch. This distinction is clinically consequential. It positions these agents as candidate harm-reduction interventions that may be relevant when immediate abstinence is neither feasible nor acceptable. They should complement, rather than displace, behavioral care and established pharmacotherapy while comparative efficacy and durability data are awaited.

## Is obesity a mechanism, a marker, or a co-indication?

Whether obesity marks treatment response or provides a co-indication remains unresolved. A direct neurobehavioral mechanism is biologically plausible. Glucagon-like peptide-1 receptors are expressed in brain regions involved in reward, and their stimulation may attenuate mesolimbic dopamine signaling, alcohol reinforcement, and cue-triggered motivation[7]. In the exenatide trial, treatment reduced alcohol-cue reactivity in the ventral striatum and septal area despite not improving the primary drinking outcome overall. This finding supports a central neurobehavioral effect but does not, by itself, establish clinical efficacy[2].

An indirect explanation deserves equal consideration. Reduced alcohol intake could result from weight loss, earlier satiety, delayed gastric emptying, altered meal patterns, or aversive gastrointestinal effects. Nausea may suppress consumption without modifying the processes sustaining AUD, while improvements in metabolic health or sleep could influence drinking through separate pathways. A third possibility is a shared reward-metabolic phenotype linking food reward, alcohol reinforcement, insulin signaling, and impulsive consumption. The exploratory exenatide benefit among participants with obesity, together with the positive obesity-restricted semaglutide trial, supports obesity as a possible response-enrichment factor but does not establish adiposity as causal^[^2,4^]^. Conversely, the oral semaglutide trial did not require comorbid obesity and found improvements in several naturalistic outcomes, suggesting that obesity may not be an obligatory response criterion[5].

Obesity should therefore be regarded as a potential response-enrichment marker and actionable co-indication, not as a mandatory biomarker. Future trials should enroll participants across body mass index (BMI) categories, model time-varying weight loss, quantify nausea and caloric intake, and use active comparators that produce similar weight loss. Neuroimaging and reward-processing biomarkers could clarify central mechanisms, while follow-up after treatment discontinuation would determine whether reduced drinking persists as gastrointestinal effects diminish or as weight returns.

## A three-axis clinical selection framework

A practical selection framework should integrate alcohol phenotype, metabolic phenotype, and treatment safety and feasibility rather than relying on the AUD diagnosis alone. First, the alcohol phenotype should resemble outcomes shown to improve in randomized trials. Patients with recurrent heavy-drinking episodes, high consumption per drinking day, prominent craving, or loss-of-control drinking may be plausible candidates, particularly when meaningful reduction is an acceptable goal after immediate abstinence has proved unacceptable or unsuccessful. Evidence is less informative for patients whose principal goal is complete abstinence, whose presentation is dominated by withdrawal, or whose drinking is sustained predominantly by severe anxiety, trauma, housing insecurity, or other social instability. In these settings, reducing reward salience cannot substitute for withdrawal management, psychiatric care, or social intervention.

Second, the metabolic phenotype determines whether treatment offers benefits beyond alcohol reduction. The strongest current rationale exists when AUD co-occurs with an independent GLP-1 RA indication, particularly for obesity or type 2 diabetes, especially in the presence of high cardiovascular risk or metabolic dysfunction-associated steatotic liver disease. Patients with overlapping metabolic and alcohol-related liver injury may be particularly relevant candidates, although evidence regarding direct AUD efficacy and hepatic safety remains insufficient in advanced liver disease. Treating 2 interacting conditions may improve the risk-benefit balance, reduce fragmented prescribing, and convert a speculative addiction intervention into evidence-based metabolic treatment with prospectively monitored alcohol outcomes.

Third, safety and feasibility can override theoretical suitability. Alcohol-related malnutrition, sarcopenia, irregular eating, recurrent vomiting, or dehydration may be worsened by appetite suppression and gastrointestinal adverse effects. A history of pancreatitis or clinically important gallbladder disease warrants individualized risk assessment, and semaglutide is not recommended in patients with severe gastroparesis. Concurrent insulin or sulfonylurea therapy also increases concern for hypoglycemia when alcohol intake and meals are unpredictable[8]. Weight trajectory, nutritional intake, muscle mass and function, hydration, glycemic therapy, and gastrointestinal symptoms should therefore be assessed before and during treatment. Advanced liver disease warrants specialist involvement because the available AUD trials do not establish safety or efficacy in decompensated cirrhosis^[^3–5^]^. Cost, medication supply, and the likelihood of sustained access also matter because interrupted treatment may not provide durable metabolic or behavioral benefit.

Most importantly, GLP-1 RAs do not treat alcohol withdrawal and must not delay evidence-based withdrawal management with benzodiazepines when clinically indicated[9]. The framework therefore identifies candidates for longer-term risk reduction, not acute withdrawal management. It is deliberately hierarchical: immediate safety first, an independent metabolic justification second, and concordance between the patient’s drinking phenotype and demonstrated trial outcomes third. It guides candidacy discussions without implying a proven individual response.

## What clinicians should do now

Current evidence supports clinical vigilance, not routine repurposing. First, clinicians should diagnose AUD, establish the treatment goal, and assess withdrawal risk before considering long-term pharmacotherapy. Second, behavioral treatment and established AUD medications, including naltrexone or acamprosate when appropriate, should remain available according to patient preference, comorbidities, and contraindications[9]. GLP-1 RA therapy should not displace effective interventions.

Third, when obesity or type 2 diabetes independently justifies a GLP-1 RA, clinicians should screen for hazardous drinking and AUD, document baseline consumption, and present alcohol reduction as a potential benefit rather than a guaranteed response. Fourth, follow-up should assess heavy-drinking days, drinks per drinking day, craving, alcohol-related consequences, and WHO risk drinking level alongside weight, nutritional status, glycemic therapy, hydration, and adverse effects. Objective biomarkers may complement but not replace patient-reported outcomes.

Fifth, off-label prescribing solely for AUD should be confined to specialist care or clinical trials until larger, longer, comparative studies establish efficacy and durability. Real-world evidence strengthens plausibility but not causality. Swedish registry data associated semaglutide use with fewer AUD-related hospitalizations[10]. A large U.S. electronic health record study found lower incidence and recurrence of AUD among semaglutide recipients[11]. Both estimates remain vulnerable to confounding, treatment selection, differential engagement, and healthcare access.

## Conclusion

GLP-1 RAs have progressed beyond being an incidental observation of weight loss, but evidence does not justify classifying them as universal addiction treatments. Their near-term role is plausibly phenotype-enriched and goal-specific: reducing heavy-drinking intensity and cardiometabolic risk in selected patients when AUD coexists with obesity or type 2 diabetes. This opportunity should not obscure uncertain durability, limited comparative evidence, or the importance of established AUD care. The next generation of trials must determine not merely whether GLP-1 RA therapy works, but for whom, through which mechanism, against which comparator, at what dose, and whether benefit persists after treatment discontinuation.

## Data Availability

No new datasets were generated or analyzed for this editorial. All evidence discussed in the article was derived from previously published sources cited in the reference list.

## Research registration unique identifying number (UIN)

Not applicable. This article is an editorial and does not report an original human study or a systematic review requiring prospective registration.

## References

[1] Richards JR Dorand MF Royal K. Significant decrease in alcohol use disorder symptoms secondary to semaglutide therapy for weight loss: a case series. J Clin Psychiatry 2023;85:23m15068. 10.4088/JCP.23m15068 38019594

[2] Klausen MK Jensen ME Møller M. Exenatide once weekly for alcohol use disorder investigated in a randomized, placebo-controlled clinical trial. JCI Insight 2022;7:e159863. 10.1172/jci.insight.159863 PMC9675448 36066977

[3] Hendershot CS Bremmer MP Paladino MB. Once-weekly semaglutide in adults with alcohol use disorder: a randomized clinical trial. JAMA Psychiatry 2025;82:395–405. 10.1001/jamapsychiatry.2024.4789 PMC11822619 39937469

[4] Klausen MK Justesen SK Pedersen JN. Once-weekly semaglutide versus placebo in patients with alcohol use disorder and comorbid obesity: a randomised, double-blind, placebo-controlled trial. Lancet 2026;407:1687–98. 10.1016/S0140-6736(26)00305-3 42070571

[5] Schacht JP Sakai JT Raymond K. Oral semaglutide for alcohol use disorder: a randomized clinical trial. Am J Psychiatry 2026;183:636–45. 10.1176/appi.ajp.20260003 PMC13567346 42522065

[6] Witkiewitz K Anton RF O’malley SS. Reductions in World Health Organization risk drinking levels as a primary efficacy end point for alcohol clinical trials: a review. JAMA Psychiatry 2025;82:1246–54. 10.1001/jamapsychiatry.2025.2508 PMC12739655 40960803

[7] Jerlhag E. GLP-1 receptor agonists: promising therapeutic targets for alcohol use disorder. Endocrinology 2025;166:bqaf028. 10.1210/endocr/bqaf028 PMC11879929 39980336

[8] Novo Nordisk Inc. Wegovy (semaglutide) [prescribing information]. Revised February 2026. Accessed 19 August 2026. https://www.accessdata.fda.gov/drugsatfda_docs/label/2026/215256s033lbl.pdf

[9] Wood E Bright J Hsu K. Canadian guideline for the clinical management of high-risk drinking and alcohol use disorder. CMAJ 2023;195:E1364–E1379. 10.1503/cmaj.230715 PMC10581718 37844924

[10] Lähteenvuo M Tiihonen J Solismaa A. Repurposing semaglutide and liraglutide for alcohol use disorder. JAMA Psychiatry 2025;82:94–98. 10.1001/jamapsychiatry.2024.3599 PMC11561716 39535805

[11] Wang W Volkow ND Berger NA. Associations of semaglutide with incidence and recurrence of alcohol use disorder in real-world population. Nat Commun 2024;15:4548. 10.1038/s41467-024-48780-6 PMC11133479 38806481

